# *Plasmodium falciparum msp2* Genotypes and Multiplicity of Infections among Children under Five Years with Uncomplicated Malaria in Kibaha, Tanzania

**DOI:** 10.1155/2015/721201

**Published:** 2015-12-06

**Authors:** W. Kidima, G. Nkwengulila

**Affiliations:** College of Natural and Applied Sciences, Department of Zoology and Wildlife Conservation, University of Dar es Salaam, P.O. Box 35064, Dar es Salaam, Tanzania

## Abstract

Genetic diversity of* Plasmodium falciparum* may pose challenges in malaria treatment and prevention through chemotherapy and vaccination. We assessed* Plasmodium falciparum* genetic diversity and multiplicity of infection (MOI) of* P. falciparum* infections and sort relationship of parasitaemia with* P. falciparum msp2* genotypes as well as with the number of infecting clones. The study was carried out in Kibaha, Tanzania. Ninety-nine children under five years with uncomplicated malaria were recruited. Genetic diversity was analyzed by genotyping the* msp2* gene using PCR-Restriction Fragment Length Polymorphism. Thirty-two different* msp2* alleles were obtained. The* msp2* 3D7 allelic frequency was higher (48.1%) and more prevalent than FC27 (27.3%) (*p* < 0.05). Twenty-four percent of the infections were mixed alleles. The individuals with FC27 had high parasitemia compared to those with 3D7 alleles (*p* = 0.038). The mean MOI was low (1.4 clones, 95% CI 1.2–1.5). The* P. falciparum* population among children at Kibaha is composed of distinct* P. falciparum* clones, and parasites having 3D7 are more frequent than those with FC27 alleles. Individuals with parasite having FC27 alleles have high parasite densities suggesting that parasites with FC27 alleles may associate with severity of disease in Kibaha. Low MOI at Kibaha suggests low malaria transmission rate.

## 1. Introduction

Genetic diversity within* P. falciparum* is a major characteristic and a factor by which the parasites survive the hosts' immune responses. It results from allelic polymorphism, recombination, chromosome rearrangements, and antigenic variation [1]. Experimental crossing of genetically distinct cloned lines of this species by Walliker et al. (1987) [2] established that genetic recombination occurs during meiosis, creating parasites of novel genotypes. Genetic diversity of malaria parasites represents a major issue in understanding several aspects of malaria infection and disease-transmission dynamics and has bearing on malaria vaccine development. In endemic areas, individuals are continuously exposed to infective mosquito bites. Due to high malaria transmission intensity in some parts of Tanzania, an individual may be infected with genetically diverse clones that may be related to the risk of clinical malaria [3–6]. It is thus imperative to assess diversity of strains/clones in blood samples among individuals in malarial areas.

The mechanisms controlling the genetic diversity within the parasite genome are many and complex. Studies have shown extensive polymorphism in some key parasite antigen during the parasites asexual blood stage (reviewed in [7]). The extensive degree of polymorphism noted in many surface antigens contributes to immune evasion and aids parasite pathogenesis [8]. It has been proposed that polymorphism of blocks 2 and 3 of the gene coding for the* msp1*,* msp2*, and* glurp* of* P*.* falciparum* can be used as genetic markers for genotyping of field parasite populations [9]. Studies have shown that the locus of the* msp2* gene of* P. falciparum* is extremely polymorphic [10] and therefore most informative. Understanding genetic diversity and multiplicity of infection is important in understanding the epidemiology of* Plasmodium falciparum* infections in endemic areas.

In the present study, Polymerase Chain Reaction- (PCR-) Restriction Fragment Length Polymorphism (RFLP) was used to define the diversity and distribution of* msp2* of alleles among symptomatic* P. falciparum* infections and assess the multiplicity of infection among children with uncomplicated malaria. The* msp2* genotypes and multiplicity of infection were related to peripheral parasitaemia.

## 2. Materials and Methods

**Figure 1:**
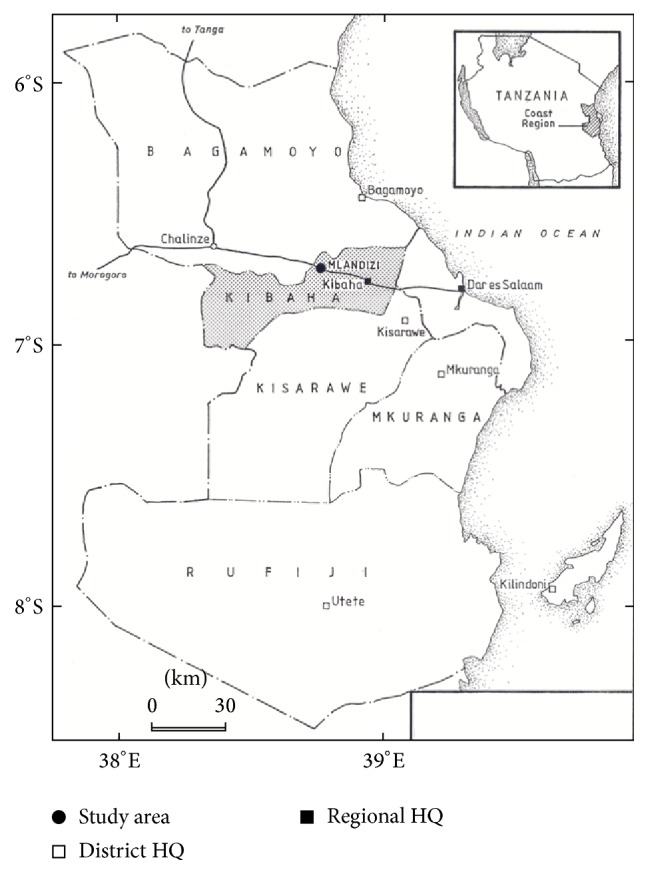
Location of the study area.

This study was carried out at Mlandizi, Kibaha, in the Coastal Region, Tanzania (Figure 1). Mlandizi was an ideal site for this study, as malaria transmission is perennial with a mean annual entomological inoculation rate of over 200 infective bites per person per year [11]. In this region,* P*.* falciparum* is the dominant malaria species. Malaria transmission in Kibaha peaks towards the end of the long and short rains.

### 2.1. Blood Sample Collection

*Plasmodium falciparum* isolates were collected during a monitoring programme of Sulphadoxine-Pyrimethamine (SP) to establish its efficacy using WHO 14-day protocol. One hundred and nineteen (119) children aged 6–59 months attending Mlandizi Health Centre were enrolled during the 2002 rainy season. Inclusion criteria were monoinfection with* P. falciparum* with at least 300 parasites/*μ*L, no intake of antimalarials or sulphonamide-based drugs during the previous 4 weeks, absence of severe illness, presence of axillary temperature of  ≥37.5°C but <39.5°, no history of allergic reactions to the administered drug, and willingness to give consent to participate. Infected blood was taken from children presenting with nonsevere malaria at day 0, before drug administration. Upon presentation to day 0, thick and thin blood smears were collected by finger prick and stained with Giemsa for microscopic parasite identification and quantification. In the thick smear, parasites were counted against 200 white blood cell count, assuming this to be 8000/*μ*L. Parasite density was recorded as the number of parasites per 200 white blood cells (WBC). Taking 8000 WBC as the conversion factor, densities were converted to the number of parasites/*μ*L of blood. The parasites density was expressed as the number of parasites/*μ*L. Assessment of the treatment response was based on the WHO 14-day* in vivo* protocol.

Ethical approval was given by the National Institute for Medical Research, clearance ref. no. NIMR/HQ/R.8a/Vol.1X/107. Verbal informed consent was obtained from village leaders and written consent was obtained from all parents or guardians.

### 2.2. DNA Extraction and Genotyping of* msp2* Gene

At enrolment, finger prick blood was also spotted on 3 mm Whatman filter paper (Whatman International Ltd., Maidstone, United Kingdom), dried, and kept separately to avoid contamination at room temperature. Parasite genomic DNA was extracted from 116 samples by chelex extraction as described elsewhere [12]. Briefly, sample parasites were boiled in the presence of chelex, heavy metal chelator. The blotted filter paper of about 3 mm^2^ was cut and placed in 1.5 mL capacity microfuge tubes containing 1 mL of PBS (pH 7.4) and 50 *μ*L of 10% saponin and incubated at 4°C overnight. The tubes were then centrifuged to aspirate the reddish PBS/saponin. Thereafter, 1 mL of PBS was added to each tube inverted each time and then incubated at 4°C for two hours. The solution was then centrifuged for two minutes and again the aspiration of the liquid was done, drying the filter paper by removing the liquid. 100 *μ*L of sterile water was added to each tube together with 50 *μ*L of 20% chelex. Chelex was then transferred to the microfuge tube using a yellow tip, with its tapered end clipped off, inverting the chelex solution after every transfer. Then, the parasite DNA was extracted by incubating the tubes for 20 minutes on a 100°C heat block vigorously vortexing each sample every two minutes.

After incubation, tubes were centrifuged for 2 minutes and then 10 minutes at 14,000 rotations per minute to remove chelex resin, leaving the parasite DNA in the supernatant. The parasite DNA was then stored in microfuge tubes at −20°C, for PCR amplification. To genotype different forms of* P. falciparum* in these samples, the polymorphic repetitive regions of the merozoite surface protein 2 (*msp2*) were amplified by a nested PCR assay [13] using oligonucleotide primer pairs specific for* P. falciparum* and alleles determined by RFLP. The initial amplification using the outermost 5′ and 3′ primers, S2, and S3 (Table 1) was carried out on 20 *μ*L reaction volume containing Ix PCR Buffer-MgCl_2_, (Invitrogen), 1.5 mM MgCl_2_, 125 *μ*M mixture of deoxynucleoside phosphates, 0.25 *μ*M of primer pair (S2/S3), 0.02 U of Taq DNA polymerase, and 5 *μ*L of genomic DNA. The PCR reaction involved 30 amplification cycles composed of initial denaturation at 94°C for 5 minutes, a denaturing step at 94°C for 1 minute, followed by annealing at 55°C for 2 minutes, and an extension step for 2 minutes at 72°C. In the second round amplification reaction, 2 *μ*L of DNA amplicon from the primary PCR was used as a template in a 30 *μ*L reaction volume containing same reaction mix as in the primary reaction, using nested conserved region 5′ and 3′ primers S1 and S4 (Table 1) to amplify the central region of* msp2* gene. Same cycling parameters were used as in the primary PCR. The nested PCR products were then monitored on 2% agarose gel before restriction digestion. A* Hinf* I (New England Biolabs) restriction digest was performed to identify* msp2* alleles in parasite isolates. Digestion was carried out overnight and digestion products were run on 10% PAA gel (Q-Bio gene, Canada) using 1.5 mm spacers, stained with ethidium bromide, then observed under UV light, and photographed. A 1 Kb ladder was used as a DNA size was used as a marker on all gels.* Hinf* I restriction digestion of a nested PCR product of FC27-type alleles produced two conserved fragments of 115 and 137 base pairs (bp) and a repeat unit of 96 bp, which represent the 5′ and 3′ end of amplification product, respectively. Members of the 3D7-type alleles produced two conserved restriction fragments of 70 and 108 bp in length [13].

### 2.3. Data Analysis

Shannon winner index was used to calculate diversity index of* msp2* alleles. To analyze differences in the frequency of occurrence of 3D7 and FC27 alleles in sample isolates, *Z* score statistical test was used. Two-way ANOVA was used to compare mean parasite densities between children with FC27, 3D7, and mixed allele using Prism 5 (Graph Pad software). Multiplicity of infection was calculated as the highest number of alleles of each* P. falciparum* isolate.

## 3. Results

### 3.1. *msp2* Genotypes of Plasmodium Falciparum

To assess* P. falciparum msp2* genotypes 99 parasite isolate (blood samples) were analysed. Polymerase chain reaction analysis showed that (82) 81% samples were positive for* P. falciparum*. All 82 positive samples were subjected to RFLP analysis to define* msp2* genotypes/multiple infections in parasite isolates. The* Hinf* I enzyme resulted in genotype-specific pattern of bands that allow discriminating a multitude of different* msp2* alleles. Individual alleles were named according to the size of a further large* Hinf* I fragment, which varies between alleles.

**Figure 2:**
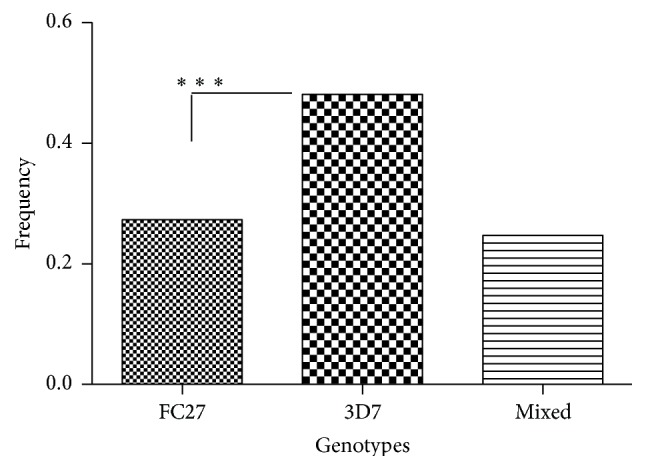
Frequency of occurrence of* msp2* genotypes among children under five years at Kibaha (*N* = 83) (^*∗∗∗*^significant difference, *p* < 0.05, *Z*-score test).

**Figure 3:**
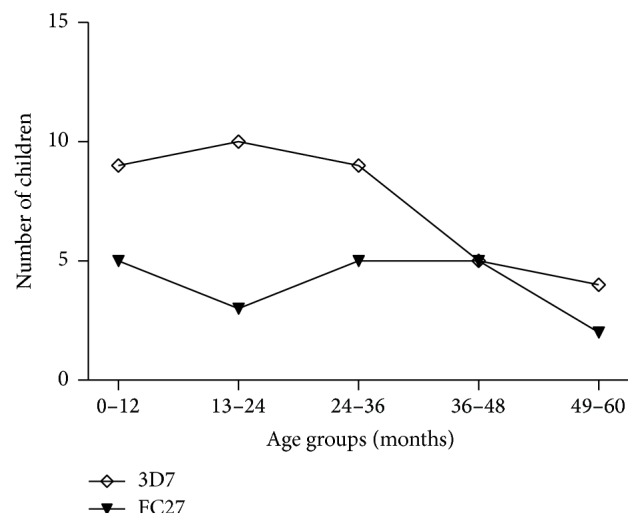
Distribution of* msp2* genotypes among children of different age groups at Kibaha at baseline (*N* = 65).

The* msp2* alleles were grouped according to similarities in their PCR-RFLP patterns. The frequency of occurrence of the two* msp2* genotypes is shown in Figure 2. Most children were infected by parasites of the 3D7 allelic family; consequently the distribution of* msp2* genotypes in different age groups showed that the 3D7 allelic types were the most prevalent in each age group (Figure 3). Overall, 51 different* msp2* alleles were detected by* Hinf* I restriction digestion of a nested PCR product. Fifteen* msp2* alleles belonged to FC27 and 17 alleles belonged to 3D7 alleles and 19 were mixed from both 3D7 and FC27 (Table 2).

**Figure 4:**
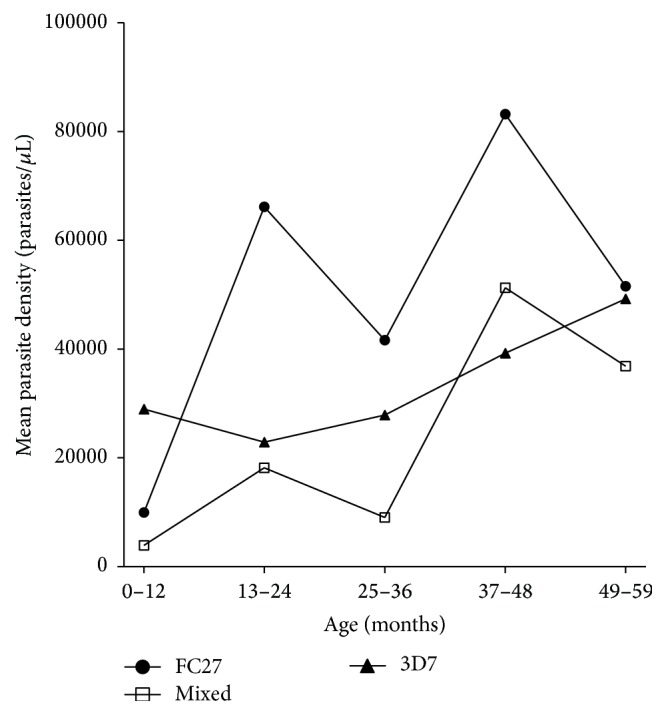
Relationship between mean parasite density and parasite genotypes in each age group (*N* = 83). Significant difference between mean parasite densities (ANOVA *F*(2, 4) = 4.2, *p* = 0.0389).

There were significant differences between mean parasite densities between individuals with FC27, 3D7, and mixed genotypes (ANOVA, *p* = 0.0389) with high parasite densities in children carrying* P. falciparum* parasites belonging to the FC27 alleles (Figure 4). Allele diversity was calculated by Shannon wiener indices (*H*′). The diversity of FC27 allele was higher (*H*′ = 1.15) than that of 3D7 (*H*′ = 1.08) but the difference was not statistically significant (Special *t*-test, df = 53, *p* > 0.05).

### 3.2. Multiplicity of Infection (MOI)

**Figure 5:**
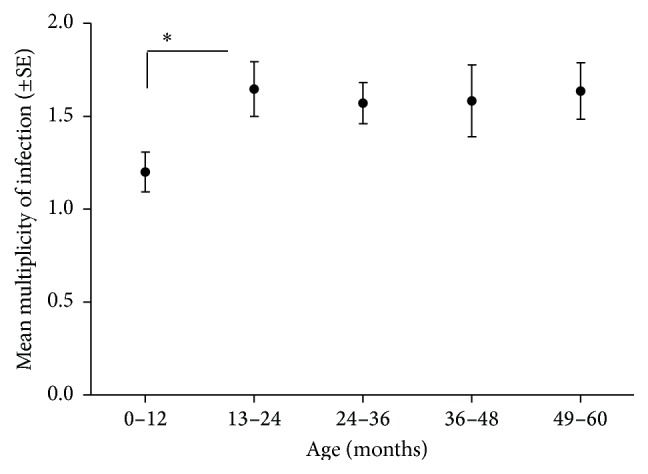
Multiplicity of* Plasmodium falciparum* infections in relation to child age at Kibaha at day 0 (*N* = 83, *t*-test, ^*∗*^ *p* ≤ 0.02).

The multiplicity of infection represents the actual number of coinfecting genotypes circulating in an individual. It was calculated as the highest number of alleles of each* P. falciparum* isolate. Most children were infected by at least two distinct parasites genotypes. Fifty percent (*n* = 38) of infected blood samples contained single infection, 47% (*n* = 36) double infections, and 3% (*n* = 2) triple concurrently infecting genotypes. The mean number of concurrent infections (i.e., MOI) was 1.4 (95% CI 1.2–1.5) (Figure 5). The highest MOI of 1.6 was observed amongst the 2-year-old children. There was significant difference between MOI in the youngest children (0–12 months) and MOI found in other age groups (*t*-test, *p* ≤ 0.05).

## 4. Discussion

The analysis of malaria-infected blood revealed by PCR-RFLP in our study has shown considerable heterogeneity among* P. falciparum* infections. We found 51 different* msp2* alleles of* P. falciparum*-*msp2* gene in blood samples collected from children under five years with clinical malaria. In this study,* P. falciparum* harboring the 3D7 parasites were more frequent than the FC27 parasites in blood samples collected from children under five years with uncomplicated malaria in Kibaha. Our findings are in agreement with other studies carried out in children with uncomplicated malaria in various malaria endemic areas [14, 15], suggesting that the 3D7 parasite strain may be the common genotype circulating in malaria endemic areas.

Although the FC27 parasites were less frequent in children under five years in Kibaha, children having these parasites genotypes had higher mean parasite density (*p* = 0.038). Association of FC27 parasites with high parasite density may suggest lack of antigen-specific antibodies amongst children due to lack of exposure and parasite immunosuppressive effects [16, 17], the conditions which might have allowed multiplication of parasites at higher densities in these infected individuals. Soulama et al. 2009 [18] observed high frequency of parasites having FC27 allelic family in severe malaria with and without anemia in children less than 5 years old in Burkina Faso, implying that the FC27 might be associated with severity of disease. Further studies in other endemic areas have to be carried out to confirm these observations.

The overall mean MOI observed in Kibaha, Mlandizi, is lower (1.4 parasite clones) compared to other holoendemic areas including some parts of Tanzania. Felger et al. (1999) [13] reported MOI of 4 at Kilombero, Tanzania, and 9.4 in Muheza, Tanga [4]. In Senegal, MOI was reported as 5 [19] and 2.1 in Kenya [20, 21]. The spatial variability in the mean MOI in malaria endemic areas may be attributed to variations in ecological parameters and anthropogenic factors. In Kibaha, being a semiurban area, transmission intensity may be low because of the lower density of Anopheles vectors. Another reason contributing to the low MOI was pointed out by Contamin et al. (1995) [22] and Magesa (1999) [4], that the effect might be an artifact caused by a single dominant clone in clinical cases overshadowing the PCR template occurring at low density. This could result in identifying few genotypes despite the presence of other genotypes in the sample. The lowest MOI in Mlandizi was observed in the youngest children (0–12). This may most probably be attributed to the short period of exposure to infection that the youngest children have had. Also protection from maternal immunity, which might have restricted the development of some genotypes, cannot be discounted.

**Figure 6:**
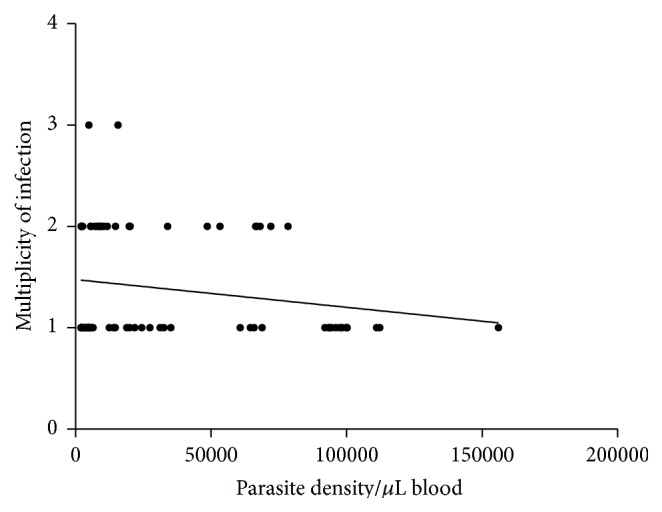
Relationship between multiplicity of infection and parasite density. Spearman rank correlation test (*r* = −0.056, CI 95%  −0.2840 to 0.1785).

There was a weak-negative association between MOI and parasite density but the relationship was not significant (see Figure 6). The weak association between MOI and parasite density might stem from the small sample size used in this study. However, the trend of low MOI in isolates with higher parasite densities suggests multiplication of a single dominant clone in the absence of high competition. Such trend has also been observed in children suffering from severe malaria in Dakar by Robert et al. (1996) [23]. Recent studies by Rono et al. 2013 [24] have implicated higher MOI with protection against severe disease in children under five, further demonstrating the role of MOI in clinical malaria.

## 5. Conclusion

We have demonstrated that the genetic diversity of* P. falciparum* isolates from children under five years at Kibaha is extensively illustrated by greater polymorphism that exists in the parasite* msp2* gene. In addition, we found that the 3D7 genotypes are more frequent than the FC27 genotypes in children under five at Kibaha. However, the FC27 genotypes are associated with higher parasite densities in children of 1 ≤ 4 years in Kibaha. Our data are important in understanding the molecular epidemiology of* P. falciparum* malaria in Kibaha, indirectly demonstrating the immune status of children with uncomplicated malaria and transmission intensity of malaria, which is spatially different in endemic areas.

## Figures and Tables

**Table 1:** Sequence of primers for amplifying *msp2* gene of *Plasmodium falciparum*.

Primer	Sequence	Primer pair
S2	5′-GAA GGT AAT TAA AAC ATT GTC-3′ (sense)	S2/S3
S3	5′-GAG GGA TGT TGC TGC TCC ACA-3′ (antisense)	

S1	5′-GAG TAT AAG GAG AAG TAT G-3′ (sense)	S1/S4
S4	5′-CTA GAA CCA TGC ATA TGT CC-3′ (antisense)	

**Table 2:** The 3D7 and FC27 *P*. *falciparum msp2* alleles recorded from children less than five years at Kibaha.

3D7 alleles	Frequency	FC27 alleles	Frequency
3D7 (200), (270)	1	FC27 (270)	2
3D7 (390)	2	FC27 (500)	1
3D7 (400)	4	FC27 (450)	1
3D7 (370)	9	FC27 (350)	1
3D7 (350)	6	FC27 (290)	2
3D7 (450)	2	FC27 (170)	2
3D7 (360), (400)	2	FC27 (250)	1
3D7 (470)	1	FC27 (330)	1
3D7 (320)	1	FC27 (137), (170)	2
3D7 (320)	1	FC27 (150), (170)	1
3D7 (300), (400)	2	FC27 (170)	1
3D7 (210)	1	FC27 (170), (260)	1
3D7 (440)	1	FC27 (150), (290)	2
3D7 (250), (400)	1	FC27 (137)	2
3D7 (520)	1	FC27 (290)	1
3D7 (170), (260)	1		
3D7 (370), (500)	1		
